# Association of physical activity and screen time with vitamin D status among children with disabilities

**DOI:** 10.3389/fped.2025.1558685

**Published:** 2025-05-30

**Authors:** Wen Wang, Zhe Wang, Haixia Sun, Guowei Li, Su Liu, Zheng Xue

**Affiliations:** ^1^ Shanghai Municipal Hospital of Traditional Chinese Medicine, Shanghai University of Traditional Chinese Medicine, Shanghai, China; ^2^ Department of Pediatrics, Jinan Massage Hospital, Jinan, China; ^3^ College of Acupuncture and Tuina, Shandong University of Traditional Chinese Medicine, Jinan, China; ^4^ Management Office, Jinan Qimingxing Children’s Rehabilitation Center, Jinan, China; ^5^ Department of Rehabilitation, Xiong’an Xuanwu Hospital, Xiongan, China

**Keywords:** NHANES, children with disabilities, physical activity, screen time, vitamin D

## Abstract

**Background:**

Physical activity (PA) and screen time (ST) may influence vitamin D status in children with disabilities, yet their relationships remain understudied in this population.

**Methods:**

We analyzed data from 645 children with disabilities aged 3–19 years. PA and ST were categorized into quartiles. Multiple linear regression models were used to examine associations between PA, ST, and vitamin D levels, adjusting for demographic and clinical characteristics.

**Results:**

Higher PA was positively associated with vitamin D levels (*β* = 0.984, 95% CI: 0.388–1.58, *p* = 0.003) in fully adjusted models. Participants in the highest PA quartile had significantly higher vitamin D levels compared to the lowest quartile (*β* = 6.884, 95% CI: 2.736–11.031, *p* = 0.003). Conversely, ST showed an inverse association with vitamin D levels (*β* = −0.8, 95% CI: −1.414 to −0.186, *p* = 0.015), with the highest ST quartile showing significantly lower vitamin D levels compared to the lowest quartile (*β* = −8.098, 95% CI: −13.318 to −2.877, *p* = 0.005). Males were more likely to engage in high PA (66.31%), and both PA and ST showed significant age-related patterns.

**Conclusions:**

Our findings reveal clear links between physical activity, screen time, and vitamin D levels in children with disabilities. Promoting physical activity and reducing screen time may be effective strategies to improve vitamin D status in this population.

## 1 Introduction

Disability affects approximately 240 million children and adolescents worldwide, representing a significant global public health concern (1). Disability may manifest at different functional levels and across multiple functional domains, including vision, hearing, mobility, cognition, and others. The Washington Group (WG) questions contain multiple response options that can be used to identify one or more indicators of overall disability status. Despite the well-documented benefits of physical activity for this population, ensuring adequate physical activity levels remains a critical challenge (2).

Physical activity behaviours are primarily evaluated through two key components: physical activity (PA) and sedentary time (ST) (3). Physical activity refers to any bodily movement produced by skeletal muscles that requires energy expenditure (4). Sedentary behavior is defined as any waking behavior characterized by an energy expenditure ≤1.5 metabolic equivalents while in a sitting, reclining, or lying posture. Recent global data indicate that excessive sedentary time has become increasingly prevalent among children and adolescents worldwide (5).

Emerging evidence suggests a potential relationship between physical activity patterns and vitamin D status, a crucial nutrient for optimal development. Vitamin D is primarily obtained through dietary intake and sunlight exposure. Beyond its well-established role in maintaining bone health (6), vitamin D has been increasingly recognized for its influence on non-skeletal health aspects, including fertility and reproductive function (7), cardiovascular disease prevention (8), and immune system regulation (9). The significance of maintaining adequate vitamin D levels is particularly pronounced during childhood and adolescence due to rapid growth and development. However, vitamin D deficiency remains a prevalent public health concern among children (10).

Children and adolescents with disabilities face unique barriers to engaging in physical activity, potentially leading to even lower activity levels compared to their typically developing peers.

Although studies by Song (11), Ouyang et al (10), have demonstrated correlations between vitamin D, PA, and ST in typically developing children. However, the reduced motor abilities and limited exercise environments of children with disabilities raise questions about whether these relationships exist in this special population, which warrants further exploration. This study aims to comprehensively investigate the associations between these factors in this vulnerable population to inform evidence-based interventions and appropriate exercise guidelines, ultimately improving physical fitness and overall well-being for children with disabilities.

## 2 Methods

### 2.1 Study population

Data for this study obtained from the National Health and Nutrition Examination Surveys (NHANES). This is a cross-sectional survey administered by the National Center for Health Statistics(NCHS) and Centers for Disease Control and Prevention. NHANES is a nationally representative, population-based survey for assessing adult and child health and nutritional status in the US. To assemble a sample of participants who were representative of the civilian noninstitutionalized U.S. population, a repeated 2-year cycle survey with a complex multistage probability sampling design was used (12). The data collection protocols are approved by the NCHS Ethics Review Board, and all survey participants provide informed consent prior to being interviewed and examined.

**Figure 1:**
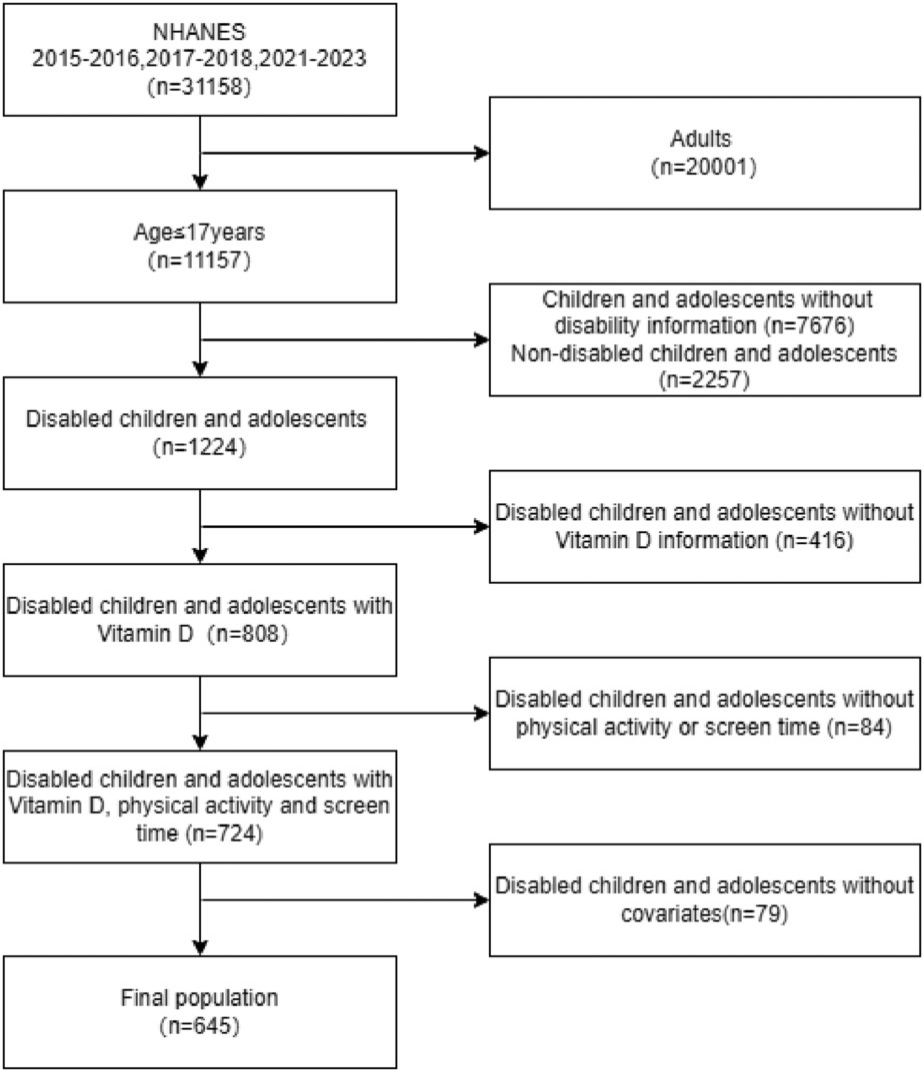
Flow diagram of participant selection from the NHANES 2015–2016, 2017–2018, and 2021–2023 cycles.

This study conducted a retrospective analysis using survey data from three cycles: 2015–2016, 2017–2018, and 2021–2023. Other waves of NHANES were not utilized due to the absence of a definition for children with disabilities in those waves. A total of 31,158 participants were included in the surveys from these years, of which 1,224 were children and adolescents with disabilities aged ≤17 years. Among these children and adolescents, 808 had complete data on vitamin D levels, physical activity (PA) time, and screen time (ST). Of these, 645 participants had complete data on covariates including age, sex, race/ethnicity, family income, BMI, asthma, and anemia. The sample selection process is illustrated in Figure 1.

Disability was defined based on the Disability Questionnaire (DLQ) in the NHANES 2015–2016 and 2017–2018 cycles. An individual was considered disabled if they answered “yes” to any of the following questions: “Have serious difficulty hearing?”, “Have serious difficulty seeing?”, “Have serious difficulty concentrating?”, “Have serious difficulty walking?”, or “Have difficulty dressing or bathing?”. In the 2021–2023cycle, the definition of disability was based on the “with disabilities” option of the Disability Indicator (FNDCDI) in the Child Functioning Module (CFM) questionnaire. Participants were classified as “with disabilities” if they were reported to have “a lot of difficulty” or “cannot do at all” for at least one of the 14 questions across 11 domains. These domains covered a comprehensive range of functional areas: seeing, hearing, mobility, self-care, communication, learning, remembering, concentrating, accepting change, controlling behavior, making friends, anxiety, and depression. It is important to note that the FNDCDI encompasses all the content previously included in the DLQ.

### 2.2 Exposure

PA and ST were measured with a self-reported questionnaire during the household interview. PA was assessed using a single question, where participants reported the number of days in the past week they engaged in physical activity for more than 60 min. Based on the 24-Hour Movement Guidelines (13) and clinical practice standards, PA was categorized into 4 groups: very low activity (0–2 days), low activity (3–4 days), medium activity (5–6 days), and high activity (7 days).

ST was measured differently across survey cycles. In the 2015–2016 and 2017–2018 cycles, ST was calculated as the sum of responses to “Hours watch TV or videos past 30 days” and “Hours use computer past 30 days”. In the 2021–2023 cycle, ST was based on the response to “hours usually spend playing with a smartphone or computer, watching TV or movies, or playing video games”. Following recommendations from the 24-H Movement Guidelines and clinical practice, ST was categorized into 4 groups: very low (0–2 h), low (3–5 h), medium (6–8 h), and high (≥9 h).

### 2.3 Outcome

The serum vitamin D level (nmol/L) was determined in this study by summing the 25-hydroxyvitamin D2 and 25-hydroxyvitamin D3 levels. Ultrahigh-performance liquid chromatography–tandem mass spectrometry was utilized for the quantitative detection of vitamin D levels. The laboratory procedure manual outlines the methodologies adopted for collecting, transporting, storing, and analyzing vitamin D samples (14).

### 2.4 Covariates

Covariates were selected *a priori* based on biological plausibility of being a confounder in the relationship between the exposure and primary outcome. Demographic characteristics such as age, sex, and race (grouped as Hispanic, non-Hispanic) were included. Ratio of family income to poverty level, and health insurance coverage were included as socioeconomic covariates. There are health-related data such as BMI, anemia, asthma, and passive smoking. BMI was classified based on the Centers for Disease Control and Prevention (CDC) “BMI-for-age charts, 2–20 years, by sex and age” growth charts. Age in months at examination was used to match the corresponding age in months from the BMI growth chart data, separately for males and females. BMI categories were defined as follows: Underweight, Normal weight, Overweight, and Obese. The age-and sex-specific 5th, 85th, and 95th percentiles of the growth charts are usually used as cutoff criteria for children and adolescents. Cutoff criteria are based on the Centers for Disease Control (CDC) growth chart “BMI-for-age charts, 2–20 years, by sex and age” (15).Anemia status was determined based on the response to the question “Taking treatment for anemia during the past 3 months”. Asthma status was assessed using the question “Ever been told you have asthma”.

### 2.5 Statistical analysis

To account for the complex, multistage probability sampling design of NHANES, we employed the Survey package in R for data extraction and analysis. This package allows for the incorporation of sample weights, as well as the use of masked variance units for strata and primary sampling units (PSUs) provided in the NHANES dataset. These techniques ensure that our results are representative of the U.S. civilian noninstitutionalized population.

For descriptive statistics, continuous variables were presented as mean ± standard deviation and compared using independent *t*-tests, while categorical variables were expressed as frequencies and percentages and compared using chi-square tests. To examine the associations between physical activity (PA), screen time (ST), and vitamin D levels, we conducted logistic regression analyses. Separate regression models were constructed for PA and ST with vitamin D as the dependent variable. Models were adjusted for potential confounding variables including sex, age, race/ethnicity, household income, body mass index (BMI), asthma status, and anemia status. A *p*-value < 0.05 was considered statistically significant.

## 3 Results

### 3.1 Subjects and demographic characteristics

**Figure 2:**
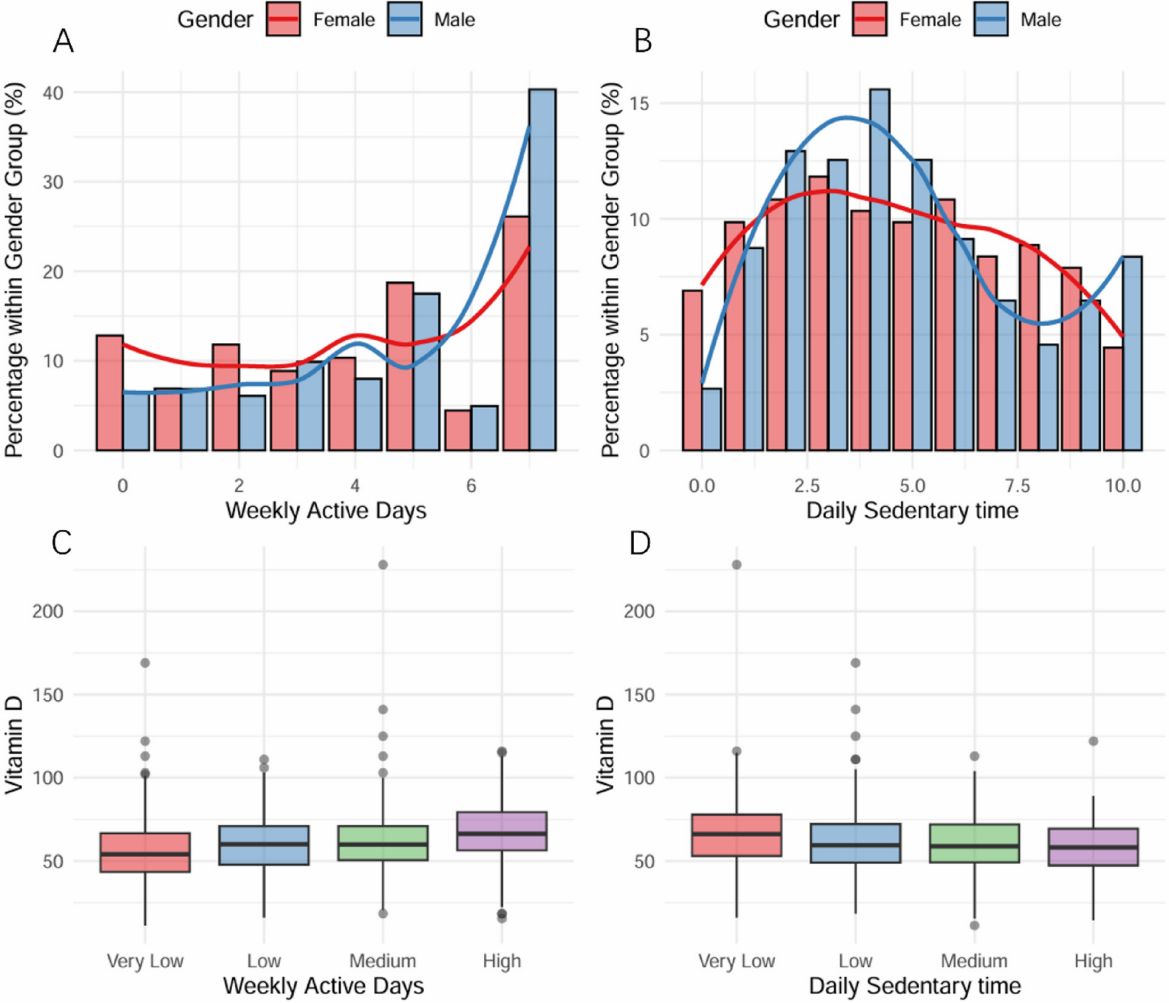
Physical Activity, Screen Time, and Vitamin D Status Among Children and Adolescents with Disabilities. **(A)** Distribution of weekly days with >1 hour physical activity by gender. The highest percentage was observed in the 7-days group, with females predominating in the ≤5 days group and males in the ≥6 days group. **(B)** Daily screen time distribution by gender. The 6-hour duration showed peak frequency, with males exceeding females in ≤6 hours groups. **(C)** Relationship between physical activity levels and Vitamin D levels, demonstrating a positive association. **(D)** Association between daily sedentary time (categorized as Very Low to High) and Vitamin D levels, showing an inverse relationship.

A total of 645 participants were included in the current study. Baseline participant characteristics, stratified according to physical activity and screen time levels, are shown in Table 1 and Figure 2. The participants with higher physical activity levels were more likely to be male (66.31% in high PA group vs. 43.63% in very low PA group, *p* = 0.003) and younger (mean age 9.88 ± 3.28 years in high PA group vs. 13.45 ± 2.98 years in very low PA group, *p* < 0.001). Similarly, participants with increased screen time were more likely to be older (13.78 ± 3.15 years in high ST group vs. 10.12 ± 3.17 years in very low ST group, *p* < 0.001).

**Table 1:** Demographic and clinical characteristics of study population (*N* = 645).

Characteristics	Over all *N* = 645(%)	Physical activity					Screen time				
		Very Low *N* = 159 (24.65%)	Low *N* = 116 (17.98%)	Medium *N* = 146 (22.63%)	High *N* = 224 (34.73%)	*p*-value	Very Low *N* = 189 (29.30%)	Low *N* = 255 (39.54%)	Medium *N* = 129 (20%)	High *N* = 72 (11.16%)	*p*-value
Sex						0.003					0.7
Female	287 (43.47%)	88 (56.37%)	54 (40.21%)	64 (44.46%)	81 (33.69%)		86 (40.59%)	108 (42.76%)	64 (47.96%)	29 (45.95%)	
Male	358 (56.53%)	71 (43.63%)	62 (59.79%)	82 (55.54%)	143 (66.31%)		103 (59.41%)	147 (57.24%)	65 (52.04%)	43 (54.05%)	
Age	11.81 ± 3.49	13.45 ± 2.98	12.35 ± 3.24	12.17 ± 3.32	9.88 ± 3.28	<0.001	10.12 ± 3.17	11.97 ± 3.35	12.84 ± 3.40	13.78 ± 3.15	<0.001
Race						0.6				0.076	
Non-Hispanic	448 (74.80%)	107 (73.42%)	76 (71.66%)	103 (78.70%)	162 (74.56%)		131 (75.75%)	169 (71.43%)	89 (73.22%)	59 (86.52%)	
Hispanic	197 (25.20%)	52 (26.58%)	40 (28.34%)	43 (21.30%)	62 (25.44%)		58 (24.25%)	86 (28.57%)	40 (26.78%)	13 (13.48%)	
BMI						0.13					0.2
Normal weight	352 (57.37%)	90 (60.37%)	50 (48.00%)	70 (51.37%)	142 (64.57%)		121 (64.26%)	130 (53.84%)	66 (53.16%)	35 (59.28%)	
Obese	176 (24.35%)	44 (24.53%)	39 (31.16%)	47 (29.68%)	46 (16.38%)		40 (18.59%)	68 (25.05%)	42 (29.40%)	26 (28.09%)	
Overweight	98 (14.87%)	20 (11.21%)	23 (17.24%)	26 (17.50%)	29 (14.59%)		23 (14.73%)	45 (14.91%)	21 (17.43%)	9 (11.11%)	
Underweight	19 (3.42%)	5 (3.89%)	4 (3.60%)	3 (1.44%)	7 (4.46%)		5 (2.41%)	12 (6.19%)	0 (0.00%)	2 (1.52%)	
Ratio of family income to poverty	2.16 ± 1.52	2.38 ± 1.63	2.08 ± 1.47	2.27 ± 1.53	1.95 ± 1.41	0.3	2.28 ± 1.58	2.13 ± 1.53	1.89 ± 1.43	2.42 ± 1.43	0.3
Anemia						0.4					0.3
No	632 (98.06%)	157 (98.80%)	111 (96.43%)	144 (99.03%)	220 (97.57%)		187 (99.47%)	252 (98.14%)	122 (96.05%)	71 (97.50%)	
Yes	13 (1.94%)	2 (1.20%)	5 (3.57%)	2 (0.97%)	4 (2.43%)		2 (0.53%)	3 (1.86%)	7 (3.95%)	1 (2.50%)	
Asthma						0.010					0.037
No	465 (74.10%)	111 (75.47%)	70 (58.55%)	110 (78.48%)	174 (77.84%)		149 (80.24%)	190 (76.25%)	81 (66.41%)	45 (63.63%)	
Yes	180 (25.90%)	48 (24.53%)	46 (41.45%)	36 (21.52%)	50 (22.16%)		40 (19.76%)	65 (23.75%)	48 (33.59%)	27 (36.37%)	
Serum vitamin D(nmol/L)	64.74 ± 20.19	58.85 ± 20.15	63.01 ± 17.23	65.13 ± 21.80	70.26 ± 18.97	<0.001	70.28 ± 21.91	63.13 ± 20.00	63.51 ± 18.72	58.76 ± 15.48	<0.001
Covered by health insurance						0.9					0.3
No	40 (4.80%)	14 (5.43%)	6 (3.75%)	9 (4.39%)	11 (5.16%)		14 (6.04%)	16 (4.97%)	10 (5.58%)	0 (0.00%)	
Yes	605 (95.20%)	145 (94.57%)	110 (96.25%)	137 (95.61%)	213 (94.84%)		175 (93.96%)	239 (95.03%)	119 (94.42%)	72 (100.00%)	

Continuous data are presented as means ± SDs and analyzed using the Wilcoxon rank-sum test; categorical data are presented as *n* (weighted %) and analyzed using the chi-square test.

The majority of participants were non-Hispanic (74.80%) and normal weight (57.37%). No significant differences were observed in race distribution across physical activity (*p* = 0.6) or screen time (*p* = 0.076) groups. Categories showed no significant variation across physical activity (*p* = 0.13) or screen time (*p* = 0.2) levels.

### 3.2 Association between physical activity and vitamin D status

As demonstrated in Table 2, when physical activity was analyzed as a continuous variable, it was positively correlated with vitamin D levels in all three models. In the unadjusted model (Model 1), physical activity showed a significant positive association with vitamin D levels (*β* = 1.748, 95% CI: 1.152–2.344, *p* < 0.001). This positive association remained significant after adjusting for age, gender, and race/ethnicity in Model 2 (*β* = 0.997, 95% CI: 0.372–1.622, *p* = 0.003) and further adjusting for additional confounders in Model 3 (*β* = 0.984, 95% CI: 0.388–1.58, *p* = 0.003). In Model 3, we conducted subgroup analyses by survey period. PA was positively associated with vitamin D levels in both the 2015–2016 and 2017–2018 cycles (*β* = 1.21, 95% CI: 0.48–1.94, *p* = 0.001) as well as in the 2021–2023 cycle (*β* = 2.03, 95% CI: 0.77–3.30, *p* = 0.001).

**Table 2:** Relationship between physical activity and vitamin D Status (nmol/L).

PA classification	Model 1^a^		Model 2^b^		Model 3^c^	
	*β*(95%CI)	*p*-value	β(95%CI)	*p*-value	β(95%CI)	*p*-value
Continuous in PA	1.748 (1.152, 2.344)	<0.001	0.997 (0.372, 1.622)	0.003	0.984 (0.388, 1.58)	0.003
PA categories						
Very low (0–2)	Reference					
Low (3–4)	4.167 (0.490, 7.843)	0.032	2.737 (−1.058, 6.533)	0.166	3.991 (0.34, 7.642)	0.04
Medium (5–6)	6.282 (1.738, 10.826)	0.01	3.894 (−0.515, 8.302)	0.092	4.605 (0.396, 8.814)	0.04
High (7)	11.413 (7.635, 15.191)	<0.001	6.718 (2.354, 11.083)	0.005	6.884 (2.736, 11.031)	0.003
*P* for trend	3.687 (2.52, 4.855)	<0.001	2.148 (0.874, 3.422)	0.002	2.162 (0.918, 3.405)	0.002

^a^ Model 1: no covariates were adjusted.

^b^ Model 2: age, gender, and race/ethnicity were adjusted.

^c^ Model 3: age, gender, race/ethnicity, poverty-to-income ratio, body mass index, asthma, anemia, passive smoking, health insurance.

CI, confidence interval.

When categorizing physical activity into quartiles, a positive dose-response relationship with vitamin D levels was observed, with participants in the highest physical activity category had significantly higher vitamin D levels compared to those in the lowest category (*β* = 6.884, 95% CI: 2.736–11.031, *p* = 0.003) in the fully adjusted model. A significant linear trend was observed across physical activity categories (*p* for trend=0.002).

### 3.3 Association between screen time and vitamin D status

As shown in Table 3, when ST was analyzed as a continuous variable, screen time demonstrated an inverse relationship with vitamin D levels. In the unadjusted model, screen time was negatively associated with vitamin D levels (*β*=−1.387, 95% CI: −1.93 to −0.844, *p* < 0.001). This negative association persisted after adjusting for confounders in Model 2 (*β*=−0.987, 95% CI: −1.597 to −0.378, *p* = 0.003) and Model 3 (*β*=−0.8, 95% CI: −1.414 to −0.186, *p* = 0.015). In Model 3, we conducted subgroup analyses by survey period. ST was negatively associated with vitamin D levels in both the 2015–2016 and 2017–2018 cycles (*β*=−0.72, 95% CI: −1.32 to −0.11, *p* = 0.021) as well as in the 2021–2023 cycle (*β*=−2.68, 95% CI: −3.63 to −1.74, *p* < 0.001).

**Table 3:** Association between screen time and vitamin D Status (nmol/L).

ST classification	Model 1^a^		Model 2^b^		Model 3^c^	
	β(95%CI)	*p*-value	β(95%CI)	*p*-value	β(95%CI)	*p*-value
Continuous in ST	−1.387 (−1.93, −0.844)	<0.001	−0.987 (−1.597, −0.378)	0.003	−0.8 (−1.414, −0.186)	0.015
ST categories						
Very low (0–2)	Reference					
Low (3–5)	−7.152 (−11.197, −3.108)	0.001	−4.424 (−8.203, −0.644)	0.027	−3.507 (−7.234, 0.221)	0.074
Medium (6–8)	−6.769 (−10.453, −3.085)	<0.001	−3.106 (−6.816, 0.605)	0.109	−0.673 (−4.681, 3.335)	0.744
High (9)	−11.521 (−16.486, −6.556)	<0.001	−8.536 (−13.905, −3.166)	0.003	−8.098 (−13.318, −2.877)	0.005
*P* for trend	−3.492 (−4.937, −2.047)	<0.001	−2.284 (−3.869, −0.699)	0.007	−1.842 (−3.462, −0.221)	0.033

^a^ Model 1: no covariates were adjusted.

^b^ Model 2: age, gender, and race/ethnicity were adjusted.

^c^ Model 3: age, gender, race/ethnicity, poverty-to-income ratio, body mass index, asthma, anemia, passive smoking, health insurance.

CI, confidence interval.

When categorizing physical activity into quartiles, a positive dose-response relationship with vitamin D levels was observed, with participants in the highest screen time group had significantly lower vitamin D levels compared to those in the lowest group (*β*=−8.098, 95% CI: −13.318 to −2.877, *p* = 0.005) in the fully adjusted model. A significant inverse linear trend was observed across screen time categories (*p* for trend=0.033).

### 3.4 Additional clinical characteristics

The prevalence of asthma showed significant variations across both physical activity (*p* = 0.010) and screen time (*p* = 0.037) groups. The low physical activity group had the highest proportion of asthma (41.45%), while the very low physical activity group had the lowest (24.53%). For screen time, asthma prevalence increased with higher screen time exposure, from 19.76% in the very low group to 36.37% in the high group.

The majority of participants (95.20%) were covered by health insurance, with no significant differences across physical activity (*p* = 0.9) or screen time (*p* = 0.3) groups. Anemia prevalence was low (1.94%) and showed no significant variation across physical activity (*p* = 0.4) or screen time (*p* = 0.3) categories.

## 4 Discussion

In this study, we examined the current status of physical activity and screen time among children with disabilities and their impact on vitamin D levels, with participants categorized according to the standards established in the 24-H Movement Guidelines and clinical thresholds to enhance the clinical interpretability of our findings. Our findings indicate that children and adolescents with disabilities demonstrate low physical activity participation rates, with only 24.65% meeting the WHO recommendation of 60 min of daily physical activity per week. Moreover, 42.63% of children exhibited low or extremely low activity levels, engaging in over 60 min of physical activity only 0–4 days per week. These findings align with previous research, as meta-analyses have shown that only 22% of children and adolescents with disabilities meet physical activity guidelines (16). Global studies indicate that 81% of adolescents aged 11–17 years are insufficiently active (17), with physical inactivity rates among children and adolescents with disabilities being 4.5 times higher than their typically developing peers (18).

Regarding screen time, approximately 29.3% of children met the recommended guidelines of less than 2 h of recreational screen time per day, while 31.14% were classified in the high-use group (≥6 h/day). Adolescents with physical disabilities were twice as likely to report watching television for more than 4 h per day compared to their peers without disabilities (19). Screen time has increasingly emerged as a crucial factor affecting children's physical health.

Children and adolescents with disabilities face distinct challenges compared to their peers without disabilities. Physical limitations, cognitive impairments, sensory issues, and social barriers can lead to decreased physical activity levels and increased sedentary or screen time (16). Research by Stanish et al. (20) demonstrated that children with autism spectrum disorders may experience reduced physical activity levels due to motor skill difficulties and social interaction challenges. Another study indicated that factors such as muscle weakness, spasticity, and mobility limitations in children with cerebral palsy hindered their ability to engage in active play and contributed to increased sedentary time (21). Most youth with disabilities lack equal opportunities for participation in sports and recreational activities compared to their peers without disabilities. These children have lower participation rates in school activities, and many sports facilities and venues remain inadequately accessible for children with disabilities, creating barriers to physical activity participation (19). Additionally, there is a notable lack of targeted guidelines and recommendations for appropriate physical activities specific to different types of disabilities.

Our study revealed significant gender differences in physical activity time (*P* = 0.003), with males showing higher representation in the high-activity group (66.31%) and females having greater representation in the low-activity group (56.37%). Interestingly, no significant gender differences were observed in screen time usage. The gender disparity in physical activity is well-documented, with boys typically being more active during childhood and adolescence than girls. This difference may be attributed to factors such as lower motivation, perceptual abilities, and gender disparities and biases in media representation and school physical education programs (22).

The study found that screen time increased significantly with age (*p* < 0.001), showing an inverse relationship with physical activity time. This trend aligns with previous research findings observed in both typically developing children and adolescents (23, 24). Screen time and physical activity demonstrate a compensatory relationship, where increased screen time often leads to reduced physical activity. Additionally, increased screen time before bedtime may affect sleep duration (25).As young people age, their use of electronic devices increases, driven by academic demands and screen-based activities, while parental oversight typically decreases. For adolescents with disabilities, electronic devices may play an even more significant role in their daily lives.

Our study revealed a significant positive dose-response relationship between physical activity and vitamin D levels that remained after adjustment for confounders, with the high-activity group showing the highest vitamin D levels. Conversely, screen time was inversely associated with vitamin D levels, with the high screen time group demonstrating the lowest levels, independent of confounding factors. To address the potential impact of slight differences in ST assessment methods across different NHANES cycles, we conducted subgroup analyses and found consistent conclusions across different survey periods.

Several mechanisms may explain these associations. First, both physical activity and screen time may influence vitamin D levels through their effects on adipose tissue, which absorbs vitamin D and increases inflammatory responses, thereby elevating the risk of vitamin D insufficiency and deficiency (26). However, in our study, the associations between physical activity, screen time, and vitamin D remained significant after adjusting for BMI, suggesting additional mechanisms are involved.

A key pathway may be through sun exposure, as research has identified sunlight exposure as a mediating factor between physical activity and vitamin D levels (10). Outdoor physical activity provides opportunities for cutaneous vitamin D synthesis, while excessive screen time may reduce outdoor activities and subsequent sun exposure. The timing and duration of outdoor activities may be particularly relevant, as vitamin D synthesis is most efficient during specific daylight hours.

## 5 Conclusion

A strength of our study is that we examined the relationships between both physical activity and screen time with vitamin D levels specifically in children and adolescents with disabilities, providing novel insights into this understudied population. Second, our findings demonstrate significant associations between physical activity, screen time, and vitamin D levels that persisted after adjusting for multiple confounding factors, including BMI, suggesting these relationships may operate through multiple pathways. Additionally, this is one of the few studies to consider both physical activity and screen time behaviors simultaneously in relation to vitamin D status among children with disabilities, offering a more comprehensive understanding of these interrelated factors.

Several limitations should be acknowledged. First, the cross-sectional design precludes establishing causality between physical activity, screen time, and vitamin D status. Second, reliance on self-reported measures for physical activity and screen time introduces potential recall bias. Third, although we adjusted for race/ethnicity to account for differences in vitamin D synthesis efficiency, we did not directly measure sunlight exposure patterns, which could confound the relationship between physical activity and vitamin D levels. Future longitudinal studies incorporating objective measures of both indoor and outdoor physical activity alongside detailed sunlight exposure assessments are needed to elucidate the independent effects of physical activity on vitamin D status.

## Data Availability

The raw data supporting the conclusions of this article will be made available by the authors, without undue reservation.
